# Quantifying Glucocorticoid Plasticity Using Reaction Norm Approaches: There Still is So Much to Discover!

**DOI:** 10.1093/icb/icab196

**Published:** 2021-10-19

**Authors:** Kasja Malkoc, Lucia Mentesana, Stefania Casagrande, Michaela Hau

**Affiliations:** Research Group for Evolutionary Physiology, Max Planck Institute for Ornithology, Eberhard-Gwinner-Straße, 82319, Seewiesen, Germany; Research Group for Evolutionary Physiology, Max Planck Institute for Ornithology, Eberhard-Gwinner-Straße, 82319, Seewiesen, Germany; Research Group for Evolutionary Physiology, Max Planck Institute for Ornithology, Eberhard-Gwinner-Straße, 82319, Seewiesen, Germany; Research Group for Evolutionary Physiology, Max Planck Institute for Ornithology, Eberhard-Gwinner-Straße, 82319, Seewiesen, Germany; Department of Biology, University of Konstanz, Universitätsstraße 10, 78464, Konstanz, Germany

## Abstract

Hormones are highly responsive internal signals that help organisms adjust their phenotype to fluctuations in environmental and internal conditions. Our knowledge of the causes and consequences of variation in circulating hormone concentrations has improved greatly in the past. However, this knowledge often comes from population-level studies, which generally tend to make the flawed assumption that all individuals respond in the same way to environmental changes. Here, we advocate that we can vastly expand our understanding of the ecology and evolution of hormonal traits once we acknowledge the existence of individual differences by quantifying hormonal plasticity at the individual level, where selection acts. In this review, we use glucocorticoid (GC) hormones as examples of highly plastic endocrine traits that interact intimately with energy metabolism but also with other organismal traits like behavior and physiology. First, we highlight the insights gained by repeatedly assessing an individual's GC concentrations along a gradient of environmental or internal conditions using a “reaction norm approach.” This study design should be followed by a hierarchical statistical partitioning of the total endocrine variance into the among-individual component (individual differences in average hormone concentrations, i.e., in the intercept of the reaction norm) and the residual (within-individual) component. The latter is ideally further partitioned by estimating more precisely hormonal plasticity (i.e., the slope of the reaction norm), which allows to test whether individuals differ in the degree of hormonal change along the gradient. Second, we critically review the published evidence for GC variation, focusing mostly on among- and within-individual levels, finding only a good handful of studies that used repeated-measures designs and random regression statistics to investigate GC plasticity. These studies indicate that individuals can differ in both the intercept and the slope of their GC reaction norm to a known gradient. Third, we suggest rewarding avenues for future work on hormonal reaction norms, for example to uncover potential costs and trade-offs associated with GC plasticity, to test whether GC plasticity varies when an individual's reaction norm is repeatedly assessed along the same gradient, whether reaction norms in GCs covary with those in other traits like behavior and fitness (generating multivariate plasticity), or to quantify GC reaction norms along multiple external and internal gradients that act simultaneously (leading to multidimensional plasticity). Throughout this review, we emphasize the power that reaction norm approaches offer for resolving unanswered questions in ecological and evolutionary endocrinology.

## Introduction

Hormones are blood-borne signals that, via changes in circulating concentrations, adjust an organism's phenotype to varying environmental and internal conditions. Such changes in concentrations can occur within few minutes or between seasons in vertebrates (e.g., Romero and Wingfield 2015). The field of endocrinology has made tremendous advances in understanding the environmental and internal factors that underlie such endocrine variation across populations as well as the phenotypic consequences (Romero and Wingfield 2015). However, more than 10 years ago the necessity to move from cross-sectional population-level studies to individual-level analyses of hormonal changes has been pointed out (Williams 2008; Dingemanse et al. 2010). To date, there is still confusion on how to perform individual-level analyses, with many studies adopting unsuitable experimental designs and statistical approaches and only few that employed appropriate methods.

Quantifying individual variation in endocrine phenotypes is key for addressing major ecological and evolutionary questions for two reasons. First, the assumption that individuals from one population respond to changes in environmental conditions in the same way is often incorrect, leading to a divergence in endocrine variation quantified at the population versus the individual level (Nussey et al. 2007; Dingemanse and Dochtermann 2013). Second, selection pressures act at the individual level, therefore, quantifying differences in hormonal responses among individuals is paramount for addressing questions like the costs and benefits of hormonal plasticity, how early life experiences may promote variation in plasticity, and whether endocrine plasticity allows individuals (and consequently populations and species) to keep pace with the rapid changes that characterize most environments nowadays (Williams 2008; Angelier and Wingfield 2013; Lema and Kitano 2013; Wada and Sewall 2014; Hau et al. 2016; Taff and Vitousek 2016; Guindre-Parker 2020).

The goal of this review is to show how endocrinologists could move forward if individual plasticity is quantified using a reaction norm approach that decomposes the individual-level hormonal variation into its main components (i.e., intercepts and slopes). Throughout our manuscript we use glucocorticoid (GC) hormones and avian species as our primary examples. We first summarize appropriate study designs and statistical models for using a reaction norm approach. Second, by reviewing the literature, we conclude that there is evidence for individual differences in average GC concentrations, in plastic GC variation along changes in environmental and internal conditions as well as in the covariation between these two attributes of the endocrine response of individuals. Yet, we also emphasize that only few studies thus far have used appropriate reaction norm approaches to accurately quantify individual GC variation. Third, we summarize some of the challenges associated with using reaction norm approaches and suggest solutions. Finally, we outline new research avenues that will open rewarding new fields of research to address the ecological and evolutionary forces that shape endocrine plasticity of individuals.

## Studying hormonal plasticity: the power of reaction norm approaches

Unlike fixed traits (e.g., beak size), flexible traits like hormone concentrations cannot be studied by sampling an individual once (Dingemanse et al. 2010). Instead, the ideal study design incorporates a repeated sampling of individuals along a measured gradient of interest (in environmental or internal conditions) and the use of random regression statistics to partitioning endocrine variation in a hierarchical fashion (Nussey et al. 2007; Dingemanse and Dochtermann 2013; Bonier and Martin 2016).

### From endocrine variation to endocrine plasticity: the definitions

The term “plasticity” has been debated for some time (e.g., reviewed by Whitman and Agrawal 2009), and to date there is still no consensus—especially across research disciplines; this is why authors should explicitly define the type of plasticity they study and how they measure and interpret it (Forsman 2015). In this review, we broadly define “*endocrine variation*” as any reversible change in hormone concentrations occurring at either the population or individual level in response to external (e.g., ambient temperature, humidity, predation risk, and competition) or internal conditions (e.g., energy reserves, immune activation, reproductive state, and age). Throughout the text, we specify the hierarchical level at which this variation is observed and analyzed (i.e., population or individual level). Importantly, we use the term “*endocrine plasticity*” only when referring to individual-level endocrine variation when analyzed with reaction norm approaches (i.e., the slope of the individual reaction norm; see below).

### Population-level variation: total endocrine variance

**Fig. 1:**
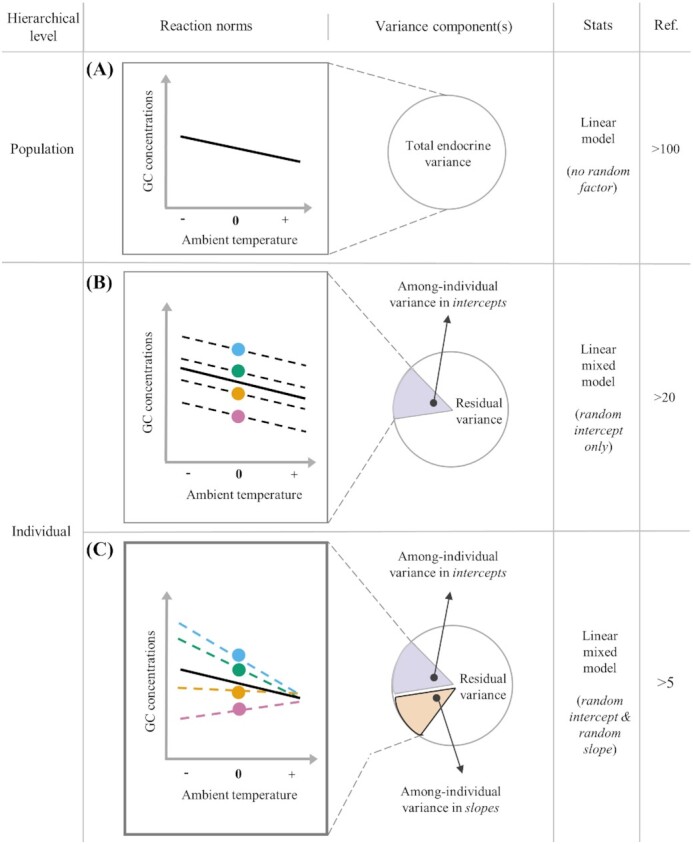
Schematic representation of phenotypic variance partitioning of glucocorticoid (GC) hormones along a mean-centered gradient in ambient temperature. Endocrine variation can be studied at different hierarchical levels (left column): population (panel **A**) and individual levels (panels **B** and **C**). A population-level decrease in GC concentrations (“total endocrine variance”) in response to increases in ambient temperature (solid lines in A–C) can result from differences in endocrine responses among individuals (broken lines in B and C indicate different individuals). When random intercepts are included in the statistical model (panel **B**), among-individual differences in average hormone titers (indicated by colored circles) can be quantified (“among-individual variance in intercepts,” purple slice). This model can also provide an imprecise measure of individual plasticity as obtained from the “residual variance” (remaining white slice in B; see text for more details). When random slopes are included in the model (panel **C**), individual differences in endocrine plasticity (represented by differences in the steepness and direction of the colored broken lines) can be accurately quantified. This “among-individual variance in slopes” (orange slice), is obtained by further partitioning the residual variation in panel B, thus resulting in smaller residual variation (remaining white slice in C). Aspects of the statistical models (presence and type of random factors) and approximate number of studies investigating GC variation at each level are provided in the “Stats” and “Ref.” columns, respectively.

At a population level, hormonal variation along an external or internal gradient can be studied using linear models, with the hormonal trait being the response variable and the gradient of interest a covariate or fixed factor (Fig. 1A). Such population-level variation, which provides information across all focal individuals, is termed “*total endocrine variance*.”

Evidence for population-level variation along a gradient in hormone concentrations is vast (see Introduction). A major weakness of this approach is that it cannot tell us whether a population-level change along the gradient is observed because all individuals change hormone concentrations in a similar way, or whether variation among individuals exists in hormonal changes (Fig. 1B and C; Nussey et al. 2007; Dingemanse and Dochtermann 2013; Niemelä and Dingemanse 2018).

### Individual-level variation: among-individual variance in intercept and slopes

In the past, individual-level variation in hormone concentrations has been quantified using different approaches. For example, a hormonal change can be calculated as the difference between the concentrations that an individual shows in two environments (Via et al. 1995; Morrissey and Liefting 2016). Alternatively, the area under the curve represents an integrated measure of an individual's hormonal response (Pruessner et al. 2003), which is usually obtained by repeatedly measuring an individual's hormone concentrations over a certain time interval. Although these methods have been appealing to endocrinologists because they can be used with only two repeatedly measured hormone samples, they have considerable limitations. First, they summarize the individual-level information contained in multiple hormone measures into a single, derived measure of individual variation for use in statistical analyses, which could be criticized as conducting “stats on stats” (Hadfield et al. 2010; Houslay and Wilson 2017). Second, and more importantly, such measures contain a mixture of biological and non-biological information and cannot indicate whether individuals differ in average hormone concentrations, in their degree of hormonal changes along a gradient, and/or in the correlation between these two attributes of their responses. For example, if selection acted only on the degree of hormonal changes in individuals, such derived measures might lead to the erroneous conclusion that endocrine responses are not under selection.

Reaction norms are powerful statistical tools to overcome the limitations outlined above (Nussey et al. 2007; Dingemanse et al. 2010; Dingemanse and Dochtermann 2013). This approach allows to decompose the total endocrine variance measured at the population level (Fig. 1A) into the two main components of an individual response: the average hormone concentrations and the degree of hormonal change (Fig. 1B and C). A detailed explanation on how to perform such models has been provided in several excellent publications (for worked examples, step-by-step tutorials and R code on how to implement such models see (Dingemanse and Dochtermann 2013; Allegue et al. 2017; Houslay and Wilson 2017). Therefore, we will only briefly discuss the two types of linear-mixed models that can be used and the information they provide. A linear mixed model with a *random intercept* term for individual identity allows to partition the population-level variation (Fig. 1A) into the “*among-individual variance in intercepts*” and the within-individual residual variance (*“residual variance”*; Fig. 1B). Variance in intercepts quantifies differences among individuals in average hormone concentrations along the environmental gradient, while the residual variance estimates how much the repeatedly measured samples differ within individuals. This within-individual residual variance (Fig. 1B) can indeed arise from endocrine plasticity (i.e., the individual adjusting hormone concentrations across samples in response to environmental fluctuation) as well as from random noise, measurement error, or additional, unmeasured environmental factors that vary across repeated samples (Westneat et al. 2015). Next, a linear mixed model that includes a *random slope* term in addition to a random intercept term allows to further partition the within-individual residual variance into the “*among-individual variance in slopes*,” which tests if individuals differ from each other in their endocrine plasticity along the gradient under study, and the remaining residual variance (Fig. 1C). From a model that includes both random intercepts and slopes it is also possible to estimate the *covariation* between intercepts and slopes (Fig. 1C), which might indicate that individuals are constrained in their endocrine responses.

Using a reaction norm approach to quantify individual-level endocrine variation confers important advantages from a statistical, biological, and evolutionary standpoint over other types of statistical approaches. First, it acknowledges the hierarchical nature of repeated measures data by decomposing—within a unique framework—the total endocrine variation into its components, allowing to quantify variation at each level simultaneously (e.g., Mentesanaetal.2019). Second, it allows to estimate individual differences in three separate, yet biologically relevant attributes of their hormonal phenotype—the intercept, the slope, and their covariation. For instance, one could discover that a significant population-level decrease in circulating concentrations of GCs in response to higher ambient temperatures (Fig. 1A) does not indicate that all individuals from that population decrease hormone concentrations similarly along this gradient (Fig. 1B and C). Instead, this overall population-level response might result from different individual patterns: individuals differing from each other in average GC concentrations (in intercepts; Fig. 1B), in the degree to which their GC titers decrease as ambient temperature increases (in slopes; Fig. 1C), and in the correlation between average concentrations and the degree of GC changes (in intercept*slope; Fig. 1C). The existence of a correlation indicates that intercept and slope may be coupled functionally (i.e., shaped by the same ecological drivers or physiological mechanisms) or genetically in individuals, suggesting that these two attributes cannot be studied independently. Such a linkage may constrain the endocrine plasticity of individuals and thus their capacity to respond to environmental changes (Nussey et al. 2007; Dingemanse et al. 2010). Furthermore, reaction norm statistics may also help to unravel why population-level endocrine patterns do not vary as predicted along a gradient (i.e., if the solid line in Fig. 1A was flat) by providing information on whether variation among- or within-individual exists but is hidden in the population-level trend.

It is worth noting that hormones often exhibit non-linear responses to environmental or internal changes (Hau and Goymann 2015). For instance, GCs can show a rapid increase when individuals are acutely exposed to major unpredictable challenges but subsequently are downregulated again to baseline concentrations. It is, therefore, plausible that non-linear reaction norms (e.g., Kingsolver et al. 2015) better describe the hormonal plasticity of individuals. However, linear reaction norms represent a valuable first step in the right direction because non-linear models are more complex (Pinheiro and Bates 2000), thus requiring larger sample sizes (see also Box 1).

Box 1. **Reaction norm approaches: challenges and solutions**

1. Using blood samples to determine hormone concentrations is ideal for studying endocrine variation at the individual level (see also Romero and Beattie 2021). However, recapturing a focal individual multiple times to take a blood sample can be difficult in studies on free-living populations. Individuals can learn to avoid traps, also resulting in sampling bias (Fusani et al. 2005). After careful validations (Goymann 2012), the use of non-invasive sampling techniques like feces, urine, hair, feathers, or hematophagous bugs (Dantzer et al. 2016) could help to repeatedly quantify endocrine traits in the same individual.
2. Individuals of certain species are not affected by being repeatedly handled for sampling (Sheldon et al. 2008), whereas in other species it can interfere with an individual's behavior and physiology during life-history stages like reproduction (Schlicht and Kempenaers 2015). One solution is to monitor behavioral and physiological responses of individuals to repeated handling to test for its impact (Houslay et al. 2019). To ameliorate adverse effects, researchers could increase the interval between sampling events or resort to non-invasive sampling techniques (see above). If sample volumes are of concern in small individuals, increasing sampling intervals could allow to collect fewer but larger samples.
3. Large sample sizes are required for reaction norm statistics, especially for the estimation of individual differences in plasticity with random slopes (Martin et al. 2011; van de Pol 2012). Moreover, testing for covariances between reaction norm intercepts and slopes is more data-hungry than estimating variation in only intercepts or slopes. Researchers should, therefore, conduct power analyses *a priori* and adjust study designs and sampling protocols accordingly. Selecting species that are particularly abundant, possibly aided by artificial breeding sites or food supplementation, that are sedentary, and/or live on islands or in ponds may permit sufficient sample sizes as well as repeated sampling of focal individuals. Multi-year and collaborative studies could also ensure the collection of required sample sizes.

## GC variation mediates phenotypic changes

In this review, we will focus on GCs because circulating concentrations in vertebrates are extraordinarily responsive to a number of environmental and internal conditions. Such variation in GC concentrations mediate major phenotypic adjustments in an array of behaviors including reproductive investment, locomotion, and foraging as well as physiological traits like energy metabolism, immune function, and cardiovascular processes (Sapolsky et al. 2000).

The regulation of circulating GC concentrations occurs primarily via the hypothalamic–pituitary–adrenal (HPA) axis in most vertebrate taxa, or the hypothalamic–pituitary–interrenal axis in fish (see Romero and Wingfield 2015 for HPA description). The overarching function of GCs is to prepare an individual for regular and predictable activities as well as support its response to, and recovery from, major unpredictable challenges. Undisturbed individuals usually maintain plasma GCs at low, baseline concentrations. Baseline GC concentrations show diel fluctuations and increase with predictable metabolic demands, for example, when environmental temperatures decrease and temporary food shortages or increased workload occur (Landys et al. 2006). When an individual experiences a major unpredictable perturbation, GCs can also increase within a few minutes to high stress-induced concentrations (Sapolsky et al. 2000). These high GC titers promote the reallocation of internal resources to recover from the threatening situation (Romero and Wingfield 2015; Romero and Beattie 2021).

Increases in GC concentrations from baseline to stress-induced levels have been studied as one kind of GC plasticity, and there exists ample evidence for individual variation in this GC “stress responsiveness” (reviewed in e.g., Hau et al. 2016; Taff and Vitousek 2016). However, since at baseline and stress-induced concentrations GCs exert their actions via different receptors, in this review we will treat them as separate traits and focus on plasticity in either baseline or stress-induced GC concentrations.

### Individual-level variation in GC concentrations

Thus far, a majority of studies on individual endocrine variation along an environmental gradient focused on among-individual variance in intercepts (Fig. 1B); that is, without analyzing whether individuals differ in their endocrine plasticity (i.e., in slopes, Fig. 1C). This may partly be explained by technical and statistical challenges (see Box 1). To date, only seven studies have applied reaction norm statistics to test for GC plasticity within a single trait of the HPA axis (most reviewed by Guindre-Parker 2020; birds: *Passer domesticus*; Lendvai et al. 2014, 2015; Baldan et al. 2021; fish: *Gasterosteus aculeatus, Poecilia reticulata*; Fürtbauer et al. 2015; Houslay et al. 2019; mammals: *Pan troglodytes, Tamiasciurus hudsonicus*; Sonnweber et al. 2018; Guindre-Parker et al. 2019). These studies indicate that this individual-level endocrine variation is explained by differences among individuals in average GC concentrations (i.e., intercept), in their degree of change along an environmental gradient (i.e., slope), or in both attributes (see also Guindre-Parker 2020). These studies are an important first step towards addressing new and fascinating questions in endocrinology: why do individuals from the same population differ in GC plasticity when exposed to the same environmental gradient? Why does an individual respond with a different GC plasticity to the same environmental gradient over time? Are individuals that are highly plastic in their GC responses also highly plastic in other phenotypic traits? In the following sections, we discuss these questions to illustrate how studying individual variation in GC plasticity using reaction norm approaches enables us to gain key insights in the ecological and evolutionary dynamics that shape hormonal processes in individuals.

## Why are not all individuals similarly plastic?

The existence of among-individual differences in slopes of GC responses was indeed supported by four out of the seven studies that used random regression models. In wild red squirrels, the slopes of fecal GC concentrations to gradients in conspecific density varied among females (Guindre-Parker et al. 2019), and in wild chimpanzees, the slopes of diel rhythms of urinary GC metabolite concentrations differed among males (Sonnweber et al. 2018). Also, captive house sparrows differed in slopes of baseline GCs when exposed to food restriction (Lendvai et al. 2014), or fluctuations in wind, heat, and food predictability (Baldan et al. 2021). Why individuals show differences in GC slopes is still unclear. We, therefore, discuss some exciting explanations for individual differences in GC plasticity that could be empirically tested.

### GC plasticity and animal personality

In many taxa, repeatable and genetically based differences among individuals in behavior are found (Koolhaas et al. 1999; Reale et al. 2010). This among-individual variation in intercepts is commonly termed “animal personality” (Reale et al. 2010). Most studies to date have linked differences in personality with population-level endocrine variation in GC stress responsiveness (i.e., the difference between baseline and stress-induced concentrations). For example, great tits (*Parus major*) that explored novel environments more slowly (“shy” personality) responded to a capture-restraint stressor with an overall faster, longer-lasting, and higher GC stress responsiveness than fast explorers (“bold” personality; Baugh et al. 2013, 2017). However, whether animal personality is related to individual GC plasticity (slopes) is still poorly understood. In wild-derived sticklebacks, personality (behavioral responses to a model predator) was associated with GC intercept but not slope (Fürtbauer et al. 2015), while in wild chipmunks (*Tamias striatus;*Montiglio et al. 2015) animal personality explained a significant portion of the residual variance in fecal GCs (i.e., the residual variance in Fig. 1B). No study to date has provided evidence for a correlational or causal link between animal personality and GC slopes; leaving open key questions as to whether personality types have emerged as consequence of individual-level GC variation or *vice versa* (Koolhaas et al. 1999).

### GC plasticity and energy availability

The plastic up- and down-regulation of circulating GC concentrations entails costs and benefits of physiological, ecological and evolutionary nature (reviews: McEwen and Wingfield 2003; Lessells 2008; Bonier et al. 2009; Romero et al. 2009; Hau et al. 2016; Taff and Vitousek 2016). Here, we will focus on energetic costs that individuals might be facing when expressing GC plasticity.

Population-level evidence indicates that increases in GCs can support metabolically challenging performances (like bird flight or reproduction; e.g., Rivers et al. 2017; Casagrande et al. 2018), which can increase fitness. However, rapid adjustments in GC concentrations may require energy to fuel the underlying physiological machinery (including tissues, receptors, and enzymes involved in the HPA axis activation; Lessells 2008; Hau et al. 2016; Taff and Vitousek 2016) as well as the resulting changes in cellular, tissue, and organismal functioning. This suggests that individuals might pay some energetic cost when expressing GC plasticity, both when upregulating and decreasing concentrations because these shift their metabolism between catabolic and anabolic states. Thus, when individuals experience energy constraints, i.e., when their energy expenditure is high or energy intake low, their capacity to change GC levels (and adjust correlated behavioral and physiological traits) in response to environmental variation might be constrained, resulting in more shallow GC slopes. Further, individuals that are already expending some energy on maintaining high average GC concentrations, for example during the reproductive stage (Casagrande et al. 2018) might exhibit a negative correlation between the intercept and the slope of their GC reaction norm. This view posits that an individual's energetic state may affect both attributes of its GC reaction norm, but we are not aware of any study testing this idea. For instance, within a reaction norm framework the energetic state of an individual could be experimentally manipulated to test whether it impacts its GC plasticity.

Limited energy availability during early development can also result in long-lasting differences in patterns of GC secretion (Brown and Spencer 2013; Jimeno et al. 2017). One intriguing hypothesis that also still awaits empirical testing proposes that energetic deficits in early life lead to the development of less costly “routine” phenotypes, characterized by a low GC plasticity (Groothuis and Carere 2005).

## Do individuals differ in GC plasticity over time and across contexts?

Several environmental and internal factors may cause within-individual variation in GC responses, such that an individual that is repeatedly assessed along the same gradient can display divergent GC intercepts, slopes, and residual variation each time (Fig. 1C; Dingemanse and Wolf 2013; Westneat et al. 2015). Whether and why this within-individual variation in GC responses exist is largely unknown, partly because repeatedly sampling an individual for hormonal reaction norms is challenging (see Box 1), especially along exactly the same environmental gradient and in the wild (Brommer 2013). Another reason may be the complexity of the statistics, which require an additional term in the linear mixed model to analyze variation across repeated reaction norms as well as heterogeneous model residuals in contrast to the homogenous residuals commonly assumed by most models. Luckily, excellent tutorials for overcoming these limitations are available (Araya-Ajoy et al. 2015; Cleasby et al. 2015; Mitchell et al. 2016).

### Repeatability of GC plasticity

When collecting repeated reaction norms of an individual, intercepts and slopes measured during one trial could deviate from the individual's mean reaction norm attributes across all trials. This could suggest the presence of multidimensional plasticity (i.e., an unmeasured environmental gradient influencing GC concentrations; see “Does GC plasticity integrate with plasticity in other traits?”), unaccounted covariation between intercept and slope, measurement error, or true random variation (Westneat et al. 2015). Quantifying within-individual variation in GC plasticity across time or states (e.g., across life-history stages) is key for two reasons. On the one hand, this “plasticity of plasticity” in GCs might contain important biological information and might indicate that GC plasticity is an individual feature that itself can be plastic (Westneat et al. 2015). On the other hand, given that selection acts on individual attributes that are consistently expressed, quantifying the repeatability of GC slopes (Araya-Ajoy et al. 2015) is important for appraising whether GC plasticity may be shaped by selection and, if genetically determined, whether it can evolve.

A total of three studies, thus far, documented within-individual variation in GC plasticity and its repeatability. Indeed, GC plasticity was found to be moderately repeatable in captive house sparrows across breeding stages (in a time series of GC concentrations after stressor exposure; *R* = 0.50; Grant et al. 2020) and across environmental challenges (in baseline GCs in response to wind, heat, and food unpredictability; *R* = 0.61; Baldan et al. 2021). Also, wild male chimpanzees had a repeatable diel GC plasticity across years (in urinary cortisol metabolites; *R* = 0.31; Sonnweber et al. 2018).

### Repeatability of intra-individual variability

When analyzing GC responses to a known environmental gradient with reaction norm statistics, individual intercepts and slopes usually do not account for the total endocrine variation and unexplained variation remains (i.e., “residual variance,” Fig. 1C). This residual within-individual variation has long been thought to result from “random noise” and has just recently started to receive attention in light of the biological information it may contain (Westneat et al. 2015). This source of individual variation is known as “*intra-individual variability*” in behavioral ecology (Stamps et al. 2012; Biro and Adriaenssens 2013) and quantifies how predictable an individual's response is to a certain gradient. Intra-individual variability can change with age (Rivera-Gutierrez et al. 2010), but in general it represents a consistent, repeatable feature of individuals in personality studies (Stamps et al. 2012; Biro and Adriaenssens 2013). Individuals characterized by a low intra-individual variability display more predictable phenotypic responses than individuals with a high intra-individual variability.

Although many behavioral responses are mediated by endocrine adjustments, to date no study has tested whether intra-individual variability in GC responses varies across and within individuals, and if it can represent a consistent attribute of the endocrine phenotype like slopes and intercepts. For instance, the recent study that tested repeatability of baseline GC plasticity in captive house sparrows exposed to different environmental challenges (Baldan et al. 2021) could be followed up by correlational or experimental approaches to assess the repeatability of GC intra-individual variability.

## Does GC plasticity integrate with plasticity in other traits?

**Fig. 2:**
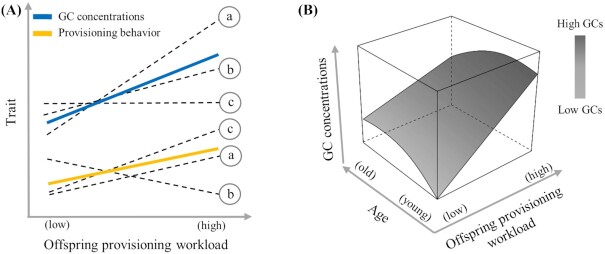
Conceptual representation of GC plasticity integration. (**A**) Multivariate plasticity: three individuals (a, b, and c) are sampled repeatedly along a gradient of offspring provisioning workload, as occurring naturally when offspring grow and require more food. Each individual is assessed for its response in two traits: GC concentrations (blue solid line) and provisioning behavior (number of feeding trips, yellow solid line). Black broken lines indicate individual reaction norms in both traits while colored solid lines indicate populationlevel reaction norms for each trait. Note that in this example the population-level correlation does not reflect the individual-level correlation as, for instance, individual b increases GC concentrations but decreases provisioning behavior, while individual c increases provisioning behavior but maintains GC concentrations unchanged. (**B**) Multidimensional plasticity: GC concentrations vary as a function of an interaction between the increased offspring provisioning workload and an individual's age. Here, GC concentrations increase as workload becomes higher, but the degree of this GC plasticity also decreases with age. Panel B is inspired by Araya-Ajoy et al. (2015).

Evolutionary biologists have long been interested in uncovering whether individuals show a similar magnitude of plasticity across different types of traits, i.e., whether some individuals respond to environmental or internal variation by always expressing highly plastic responses while others show only little plasticity (“plasticity integration”; Schlichting 1986; Husby et al. 2010). Plasticity integration can occur across different types of traits that respond to the same environmental gradient like behavior or physiology (*multivariate plasticity*, Fig. 2A). Moreover, plasticity in one trait can result from a combined response to two or more different gradients acting simultaneously (*multidimensional plasticity*), so that plasticity displayed by one individual results, for instance, from an interaction between a gradient in workload and one in age (Fig. 2B). Even though these two questions are being increasingly investigated in evolutionary and behavioral ecology (for multivariate plasticity see for instance Houslay et al. 2019; Mitchell and Houslay 2021; for multidimensional plasticity: Westneat et al. 2009, 2011; Bonamour et al. 2020), studies on plasticity integration in endocrinology have remained rare.

### Multivariate GC plasticity

By including two or more traits as response variables in a multivariate random slope model it is possible to test for correlations among reaction norms of different traits (Dingemanse and Dochtermann 2013; Araya-Ajoy et al. 2015; Careau and Wilson 2017). Specifically, this statistical approach can reveal whether correlations exist in average trait values (intercepts), trait plasticities (slopes), and residual variations.

For instance, if an individual's energetic state affects its GC plasticity, and GC plasticity influences its metabolism (see “Why are not all individuals similarly plastic?”), correlated plasticities in GCs and metabolic rates can be expected (organismal level: Jimeno et al. 2018; cellular level: Casagrande et al. 2020; and both organismal and cellular levels: Malkoc et al. 2021). However, so far only few experimental studies used a multivariate approach to test for covariation between attributes of GC reaction norms and those of other traits at the individual level. In zebra finches (*Taeniopygia guttata*), a positive correlation between GC intercepts and slopes of organismal metabolic rate and locomotor activity was found (Careau et al. 2020), while in house sparrows subjected repeatedly to food restriction the GC slope covaried negatively with the slope in body mass, but not with the slope in oxidative stress (Lendvai et al. 2014). Combining GC manipulations with multivariate statistics would establish whether GC plasticity is causally related to plasticity in metabolic traits. For instance, GCs can be experimentally increased in parental birds by shortening a few wing feathers (resulting in a greater energy expenditure when flying) during the offspring provisioning phase (Casagrande and Hau 2018). Feather-clipping could be combined in a factorial design with the administration of exogenous GCs, or GC receptor antagonists, to investigate if covariation in GCs and provisioning plasticity is based on a causal relationship (Fig. 2A, colored solid lines).

Given the scarcity of studies addressing multivariate GC plasticity within a reaction norm framework, major questions are left to be answered. For instance, do individuals from a population differ in how GC and offspring provisioning slopes are correlated (Fig. 2A; individual broken lines)? What are the proximate causes of the linkages among trait plasticities? If multivariate GC plasticity exists, does it provide fitness benefits?

### Multidimensional GC plasticity

Given the exceptional responsiveness of GCs to a variety of external and internal gradients, it is reasonable to assume that both GC intercept and slope are affected by multiple gradients simultaneously (Lema 2014; Taff and Vitousek 2016), resulting in multidimensional plasticity (Rice 2002; Westneat et al. 2019). For instance, according to the allostatic load and the reactive scope models (McEwen and Wingfield 2003; Romero et al. 2009) circulating GC concentrations result from a trade-off between the energy requirements of an individual and the amount of reserves it has stored, such that GC plasticity may be a product of the joint gradients in energy expenditure and body condition.

Multidimensional reaction norms can be assessed using random regression statistics in which two or more environmental axes are modeled in interaction, which in the case of two gradients results in 3D planes instead of 2D lines (Fig. 2B; Westneat et al. 2009; Araya-Ajoy et al. 2015). For example, parental birds might display GC plasticity when experiencing progressively higher workloads as their offspring grow and require more food, but the magnitude of this GC plasticity might decrease as individuals become older (Fig. 2B). Multidimensional reaction norms are particularly useful because researchers can quantify, within a unique model, GC intercepts and slopes along a single environmental gradient as well as “joint” intercepts and “joint” slopes (forming the 3D plane) resulting from the interactive effects of multiple environmental gradients (Westneat et al. 2009).

Natural habitats are characterized by multidimensional environmental challenges, which are becoming more extreme as climate change and urbanization are progressing (Westneat et al. 2019). Multidimensional reaction norms will allow us to address key questions, like whether the joint gradients of increased offspring provisioning workload and age (Fig. 2B) could generate inadequate individual GC plasticity to major climatic challenges that decrease food availability during the breeding season (e.g., cold spells and extended rains). This, in turn, could result in maladaptive phenotypic responses and major fitness detriments (loss of entire brood and death of parent). Correlative studies in free-living populations could be accompanied by experimental approaches in a factorial design, for example by increasing the workload of parents (e.g., by feather-clipping) of known age, while at the same time raising their energy expenditure by cooling their nest-boxes (Meijer et al. 1999).

## How does GC plasticity relate to performance and fitness?

**Fig. 3:**
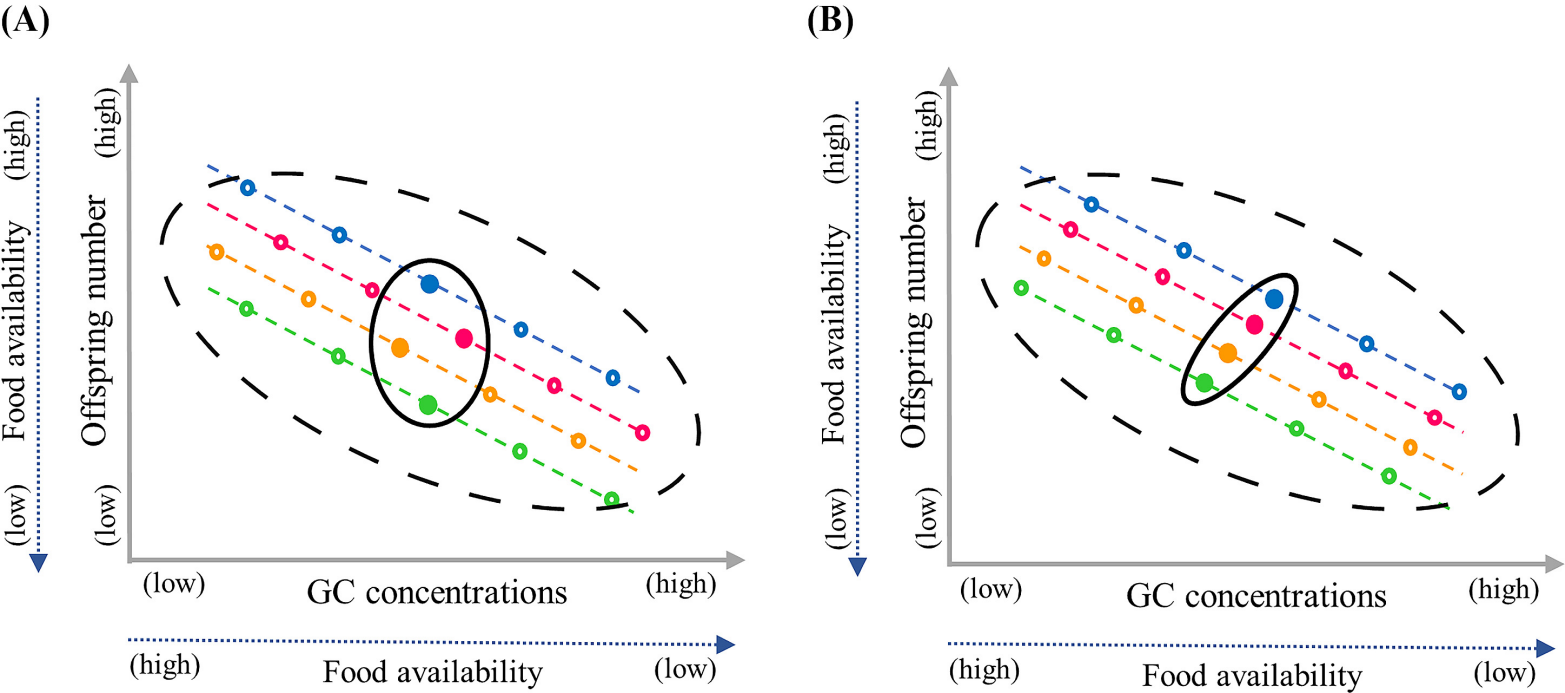
Exemplified relationships between individual variation in circulating GC concentrations (*x*-axis) and a fitness proxy (i.e., offspring number, *y*-axis), as well as their mutual dependence on an environmental gradient (food availability); inspired by Niemelä and Dingemanse 2018 and Bonier and Martin (2016). Colored circles indicate observations of the two traits for four individuals (represented by different colors) that were sampled five times along the gradient of decreasing food availability. Filled circles indicate average traits values of each individual. Population-level phenotypic correlations between the two traits (elliptic broken lines) embrace all the observations, while among-individual correlations embrace only average trait values (elliptic solid lines). Colored broken lines connect repeated measures for each individual. In both panels, when environmental conditions worsen, GC concentrations increase and offspring numbers decline. This mutual dependence of GCs and fitness on food availability drives the negative correlation observed at both the within-individual level and at the population-level. An among-individual correlation is absent in panel A, while it is present in panel B, but in an opposite direction to that observed at the within-individual and population level.

Endocrine traits can evolve when they are heritable and linked to physiological and behavioral traits that ultimately affect Darwinian fitness. A link between the HPA axis and fitness-related traits (e.g., reproductive success, survival, immunocompetence, risk-taking behaviors, and parental care) has been postulated (e.g., McEwen and Wingfield 2003; Breuner et al. 2008; Bonier et al. 2009; Schoenle et al. 2021). To date, supportive evidence for this link is mixed (Breuner et al. 2008; Bonier et al. 2009; Schoenle et al. 2021). In particular, individual-level studies have focused on GC intercepts or GC responsiveness (i.e., from baseline to stress-induced concentrations), but not on GC slopes. Therefore, a relationship between variation in GCs and fitness-related traits may be confounded by the plastic nature of GCs. That is, correlating slopes in GCs and fitness-related traits using reaction norms approaches (Bonier and Martin 2016; Niemelä and Dingemanse 2018) may dissolve this confusion. Being labile, GCs and fitness-related traits show low to medium repeatability (for GCs and fitness intercepts: on average *R* ≤ 0.4; Niemelä and Dingemanse 2018; for GC plasticity: *R* averaged over three studies ≤ 0.5; see “Do individuals differ in GC plasticity over time and across contexts?”) and consequently harbor high within-individual variation (Fig. 1B; Westneat et al. 2015; Niemelä and Dingemanse 2018; Careau and Wilson 2017). Therefore, any population-level phenotypic correlation between GCs and fitness will represent a mixture of correlations at among-individual and within-individual levels. Another issue is that GC plasticity can occur in response to the same environmental variation that can also influence fitness traits (Bonier et al. 2009; Bonier and Martin 2016; Dantzer et al. 2016). In the absence of statistical covariance partitioning it remains unclear whether GCs and fitness-related traits only covary within individuals, for example because low food availability in a given year decreases an individual's offspring number while also increasing GC concentrations (Fig. 3A). Likewise, a positive covariation of GCs and fitness-related traits only at the among-individual level could lead to false conclusions because it primarily results from individual differences in intercepts (Fig. 3B; i.e., one individual always producing more offspring than another, irrespective of food availability in a given year), and does not account for the presence of within-individual correlations, which may occur in the opposite direction.

Studies that decompose correlations between GCs and fitness-related traits at among- and within-individual levels are rare (Ferrari et al. 2013; Boulton et al. 2015; Dosmann et al. 2015). Only one of these studies found a within-individual correlation between stress-induced GC concentrations and fitness-related activity levels in alpine marmots (*Marmota marmota;*Ferrari et al. 2013). Note that although the studies above estimated within-individual correlations among labile traits, they did not statistically separate the correlations among trait plasticities (i.e., slopes) from any residual correlations as would be achieved by fitting random slopes (Fig. 1C). Indeed, to date no study has analyzed the association between both intercepts and slopes of individual GC reaction norms and attributes of fitness-related reaction norms, or/and combined it with experimental approaches to test for causality. This could be done in free-living populations, for instance, by manipulating GC reaction norm attributes (the intercept with GC implants, or the slope with cremes containing GCs; Vitousek et al. 2018) while also repeatedly measuring fitness-related traits to quantify correlated plasticities between both.

## Conclusions

Endocrine changes analyzed at the population level rarely reflect patterns at an individual level—the level at which selection acts. By using reaction norm approaches and hierarchically decomposing hormonal (co)variation into individual differences in average concentrations (intercept), in the degree of hormonal change (slope), and in the remaining residual variation, endocrinologists will greatly improve their understanding of the proximate causes and ultimate consequences of endocrine variation. Studying individual hormonal plasticity using reaction norm approaches will enable endocrinologists to resolve numerous questions that range from the neuroendocrine and molecular pathways that cause endocrine plasticity and generate among- and within-individual differences, to its ecological and evolutionary implications.
